# Hypertension: sex-related differences in drug treatment, prevalence and blood pressure control in primary care

**DOI:** 10.1038/s41371-023-00801-5

**Published:** 2023-01-19

**Authors:** Johan-Emil Bager, Karin Manhem, Tobias Andersson, Per Hjerpe, Kristina Bengtsson-Boström, Charlotta Ljungman, Georgios Mourtzinis

**Affiliations:** 1 Department of Molecular and Clinical Medicine, Institute of Medicine, Sahlgrenska Academy, University of Gothenburg, Gothenburg, Sweden; 2 Department of Emergency Medicine, Sahlgrenska University Hospital, Gothenburg, Sweden; 3 Primary Health Care, School of Public Health and Community Medicine, Institute of Medicine, Sahlgrenska Academy, University of Gothenburg, Gothenburg, Sweden; 4 Regionhälsan R&D Centre, Skaraborg Primary Care, Skövde, Sweden; 5 Department of Medicine and Emergency Mölndal, Sahlgrenska University Hospital, Gothenburg, Sweden

**Keywords:** Hypertension, Preventive medicine, Risk factors

## Abstract

Antihypertensive treatment is equally beneficial for reducing cardiovascular risk in both men and women. Despite this, the drug treatment, prevalence and control of hypertension differ between men and women. Men and women respond differently, particularly with respect to the risk of adverse events, to many antihypertensive drugs. Certain antihypertensive drugs may also be especially beneficial in the setting of certain comorbidities – of both cardiovascular and extracardiac nature – which also differ between men and women. Furthermore, hypertension in pregnancy can pose a considerable therapeutic challenge for women and their physicians in primary care. In addition, data from population-based studies and from real-world data are inconsistent regarding whether men or women attain hypertension-related goals to a higher degree. In population-based studies, women with hypertension have higher rates of treatment and controlled blood pressure than men, whereas real-world, primary-care data instead show better blood pressure control in men. Men and women are also treated with different antihypertensive drugs: women use more thiazide diuretics and men use more angiotensin-enzyme inhibitors and calcium-channel blockers. This narrative review explores these sex-related differences with guidance from current literature. It also features original data from a large, Swedish primary-care register, which showed that blood pressure control was better in women than men until they reached their late sixties, after which the situation was reversed. This age-related decrease in blood pressure control in women was not, however, accompanied by a proportional increase in use of antihypertensive drugs and female sex was a significant predictor of less intensive antihypertensive treatment.

## Introduction

Arterial hypertension remains the foremost preventable cause of cardiovascular disease and death and antihypertensive drug therapy reduces the risk of major cardiovascular events, regardless of sex, previous cardiovascular disease status and baseline blood pressure [1–3]. Men and women differ to some extent with regard to when they develop hypertension; in how they respond to drugs used in hypertension; and in both cardiovascular and non-cardiovascular health challenges [4–10].

This invited, narrative review for the special issue on Sex and Gender Differences in Hypertension explores how antihypertensive drugs can differ in effect between men and women. Next, it briefly reviews the topic of hypertension in pregnancy from a primary care perspective, because of the challenges associated with drug treatment for women who are pregnant or planning pregnancy. Finally, it reviews differences in prevalence, blood pressure control and drug class use between men and women with hypertension in primary care.

This article also presents original, real-world data to illustrate key points and to provide contrast to population-based studies regarding sex-related differences in hypertension prevalence, blood pressure control and antihypertensive drug use.

## Methods

On editorial request, this narrative review for the special issue on Sex and Gender Differences in Hypertension also features original data from patients with hypertension who were treated in primary care in the Region of Västra Götaland, Sweden. The purpose of including original data was to assess sex-related differences in hypertension prevalence, blood pressure control and antihypertensive drug class use in a large, contemporary, primary-care cohort. The patients were identified through a primary care register, QregPV, and additional data such as diagnoses and drug dispensation were acquired through national registries. The methodology has been described in detail previously and was approved by the ethical review board of the University of Gothenburg [11]. For this article, we identified all living patients with diagnosed hypertension in primary care in the Region of Västra Götaland in 2017. Controlled blood pressure was defined as <140/90 mmHg. Drug classes were defined with the Anatomical Therapeutic Chemical (ATC) Classification System: alpha blockers (C02CA); angiotensin-receptor blockers (ARBs) (C09C, C09D); angiotensin-converting-enzyme inhibitors (C09A, C09B); calcium-channel blockers (CCBs) (C07FB02, C08CA, C09BB, C09DB); beta blockers except labetalol (C07AA, C07AB, C07AG02); labetalol (C07AG01); loop diuretics (C03C); mineral-receptor antagonists (C03DA); thiazide diuretics (C03A, C03B, C03EA, C09BA, C09DA); other antihypertensives (C02A, C02D, C09XA); and statins (C10AA, C10AX, C10BA). Antihypertensive drug class use was defined as at least one dispensation of one drug in that class during 2017. The number of antihypertensive drugs used by a patient was calculated as the number of different drug classes for which prescriptions had been dispensed. Mean values and relative frequencies were calculated for continuous and categorical variables, respectively. We plotted the mean number of antihypertensive drugs used and the relative frequency of different antihypertensive drug classes by age and sex. A linear regression model with age, systolic blood pressure, sex, current smoking, history of ischemic heart disease or history of diabetes as covariates was used to analyze factors associated with the number of antihypertensive drugs used. A generalized, additive model with integrated smoothness estimation was used to visualize the age-specific relative frequency of controlled blood pressure for men and women. All analyses were performed in R (version 4.0.3) through RStudio (version 1.4.1103) [12, 13].

## Differences in effects of drugs used in hypertension

Antihypertensive drug treatment is equally beneficial in men and women and the effects of the main classes of drugs used in hypertension are the same in both sexes when comparing the risk of outcomes such as myocardial infarction, congestive heart failure, stroke, and cardiovascular and all-cause mortality [2]. However, early trials included few women and in general, women have been underrepresented in clinical trials in hypertension [14]. Women are, however, overall more likely to experience adverse effects of drugs and the drugs used in hypertension are no exception [7, 10]. There are also other clinical variables of relevance that may influence drug class choice. The following paragraphs elaborate on potential extracardiac treatment synergies and the differences in adverse effects between men and women for the main classes of drugs used in hypertension, which are summarized in Table 1.

**Table 1:** Overview of treatment synergies and differences in adverse effects.

Drug class	Cardiovascular treatment synergies	Extracardiac treatment synergies	Predisposition to adverse effect	
			Women	Men
Alpha blockers		Benign prostatic hyperplasia		
ACEi	HFrEF, ischemic heart disease	Diabetes mellitus; chronic kidney disease with proteinuria	Cough; contraindicated in pregnancy	–
ARB	HFrEF, ischemic heart disease	Diabetes mellitus; chronic kidney disease with proteinuria; migraine	Contraindicated in pregnancy	–
Beta blockers	HFrEF; ischemic heart disease; atrial fibrillation	Migraine	–	Erectile dysfunction
CCB	Chronic stable or vasospastic angina	Raynaud phenomenon	Dizziness, flushing, headache, tibial edema	–
MRA	HFrEF	Liver cirrhosis; acne vulgaris or hirsutism in polycystic ovary syndrome; transsexualism (MTF); and female androgenetic alopecia	Contraindicated in pregnancy (spironolactone)	Gynecomastia (spironolactone)
Thiazides		Osteoporosis; nephrolithiasis	Hyponatremia, hypokalemia	Gout; erectile dysfunction

Note that many of the uses above are off label and not supported by randomized, controlled trials.

*ACEi* angiotensin-converting enzyme inhibitor, *ARB* angiotensin-receptor blocker, *CCB* calcium-channel blocker, *HFrEF* heart failure with reduced ejection fraction, *MRA* mineral-receptor antagonist, *MTF* male-to-female.

### First-line drugs

#### Angiotensin-converting enzyme inhibitors and angiotensin receptor blockers

Angiotensin-converting enzyme inhibitors (ACEi) and angiotensin receptor blockers (ARB) are recommended as first-line treatment of hypertension [15, 16]. ACEi are a suitable choice in both uncomplicated hypertension and in the context of coexisting coronary heart disease, heart failure with reduced ejection fraction, diabetes mellitus or chronic kidney disease with proteinuria [16–18]. Men and women differ in regard to their risk of adverse effects, where ACEi treatment-induced cough is more than twice as likely to afflict women [6, 19].

ARB are equally well tolerated in men and women, exhibiting an adverse effect rate comparable to placebo [20]. They share indications with the ACEi, although they are now a second-line treatment in patients with heart failure with reduced ejection fraction [17, 18]. Of note, both ACEi and ARB are strictly contraindicated in pregnancy due to the risk of fetal abnormalities. Like beta blockers (see below), ARB are also effective in preventing episodic migraine, which is more than twice as common in women [21–23].

#### Calcium-channel blockers

Dihydropyridine calcium-channel blockers (CCB) are also a first-line treatment in hypertension [15, 16]. The CCB are the only first-line drug class that can be initiated without knowledge and timely follow-up of electrolyte status and kidney function, making them a convenient choice when follow-up scheduling is challenging. The vasodilating effects of CCB can cause adverse effects encompassing dizziness, flushing, headache and tibial edema, which women are more likely to experience [8, 24]. Calcium-channel blockers, especially nifedipine, are safe to use during pregnancy. They are also a suitable choice for treating Raynaud phenomenon, which is more prevalent in women [16, 25–27].

#### Thiazides

Thiazide-type and thiazide-like diuretics are also a first-line treatment in hypertension [15, 16]. Thiazide treatment is associated with a reduced risk of osteoporotic fractures, which predominantly afflict women [28]. However, observational studies have suggested that this effect may, perhaps surprisingly, be clinically more discernible in men [29–31]. One explanation to this finding could be that fracture risk in general is noticeably higher in women, and the fracture-protecting effect of thiazides might be ruled out by other more potent negative factors in women. Thiazides are also a first-line, pharmacological treatment option in patients with recurrent calcium oxalate nephrolithiasis who exhibit hyper-calciuria [32]. Calcium-oxalate stones are the most frequent cause of nephrolithiasis and men are afflicted twice as often as women [33].

Hypokalemia and hyponatremia are frequent adverse effects of thiazides, which seem to afflict women to a greater degree than men, while men are more likely to develop hyperuricemia and gout [34–36]. Men are also more likely to experience sexual dysfunction during thiazide treatment, primarily in the form of erectile dysfunction [37–39]. Thiazides are rarely used in pregnancy due to risk of decreased placental perfusion and the decreased plasma volume associated with preeclampsia [16, 27].

### Second-line drugs

#### Mineral-receptor antagonists

Mineral-receptor antagonists (MRA) do not constitute a first-line treatment in hypertension, but are important therapeutic tools in resistant hypertension, primary aldosteronism, heart failure, liver cirrhosis, and in the potassium-wasting tubulopathies of Bartter and Gitelman syndromes [16, 17, 40, 41]. Hypertension guidelines recommend MRA in resistant hypertension, where they provide twice the systolic blood pressure reduction of alpha blockers and beta blockers [15, 16, 42].

The antiandrogenic effects of spironolactone can also be harnessed in women with androgen-dependent conditions like acne and hirsutism in polycystic ovary syndrome and androgenetic alopecia, when combined estrogen-progestin oral contraceptives do not suffice [43, 44]. Spironolactone is also used in high doses to suppress testosterone secretion in the treatment of transgender women (male-to-female) [45]. Gynecomastia may occur in men treated with spironolactone, due to its antiandrogenic effects. The risk is dose-dependent and virtually all men treated with high doses of spironolactone per day will develop gynecomastia [46]. Eplerenone, which is not antiandrogenic, can be used instead [47]. In pregnancy it is advisable to avoid spironolactone, which may cause feminization of a male fetus, and opt for the safer eplerenone, if an MRA is considered necessary [48].

#### Beta blockers

Despite not being a first-line treatment in hypertension, beta blockers are frequently used in patients with hypertension. In part, perhaps, because of tradition but also because of their important role in treating manifest, hypertension-related heart diseases such as heart failure, ischemic heart disease and atrial fibrillation [16, 17, 49, 50]. Beta blockers are less effective in preventing stroke than the first-line drugs [51]. In the absence of ischemic heart disease, heart failure or atrial fibrillation, consequently, beta blockers are recommended only when blood pressure targets are not attained using first-line antihypertensive drugs and MRA [15, 16]. The antihypertensive effect of beta blockers in resistant hypertension is less than half than that of MRA [42].

Beta blockers also constitute a first-line, preventive treatment of episodic migraine [23, 52]. Labetalol, a combined alpha and beta blocker, is considered a safe treatment option when treating pregnancy-related hypertension [16, 27]. Beta blockers are associated with a slight increase in sexual dysfunction in both sexes and with erectile dysfunction in men [53].

#### Alpha blockers

Alpha blockers, such as doxazosin, can be used to treat hypertension when drugs with superior efficacy are insufficient to reach target blood pressure [15, 16]. They are not considered first-line treatment, mainly because of the results from the ALLHAT trial, in which the doxazosin arm displayed higher incidence of non-fatal cardiovascular endpoints, compared to the thiazide arm [54]. They are also inferior to MRA in resistant hypertension [16, 42]. Alpha blockers have a niche role in the management of blood pressure in the setting of a pheochromocytoma [55, 56]. Because alpha blockers relax smooth muscle tissue both in blood vessels and in the urethra and bladder neck, they are a cornerstone of medical treatment of lower urinary tract symptoms in men with benign prostatic hyperplasia (BPH) [57, 58]. Notably, doxazosin may also decrease symptoms of erectile dysfunction [59].

The most frequent adverse events of alpha blockers are headache, dizziness and postural hypotension [42]. Alpha blockers have also been linked to intraoperative floppy iris syndrome, a condition which encompasses pupillary constriction and flaccidity and prolapse of the iris, which complicate cataract surgery. As a result, guidelines recommend that men with BPH who are planning to have cataract surgery do not commence treatment with alpha blockers until cataract removal is complete [57]. As shown below in our registry data, alpha blockers are used less frequently in women, but physicians who treat women with alpha blockers for hypertension should nevertheless be aware of intraoperative floppy iris syndrome. Although alpha blockers have no known teratogenic effects, they should only be used when it is unavoidable, since experience of their use in pregnancy is scarce.

## Hypertension in pregnancy

Management of hypertension in pregnancy poses a unique challenge for both the expecting women and their physicians. This is the case when treating both gestational hypertension, which is defined as newly developed hypertension after 20 weeks of gestation, and preexisting hypertension, which is defined as hypertension that either precedes the pregnancy or has developed within the first 20 weeks of it [27]. The challenge is in part due to the narrower arsenal of available drugs, but the delicate balancing act of weighing potential advantages of treatment for the mother to potential risks for the fetus is also a major part. European guidelines from 2018 recommend initiation of pharmacological treatment when blood pressure is ≥140/90 mmHg in three groups of pregnant women: (1) those with gestational hypertension; (2) those with preexisting hypertension who develop superimposed gestational hypertension, i.e. women with preexisting hypertension who exhibit blood pressure values ≥140/90 mmHg after week 20 of pregnancy; and (3) those with signs of hypertension-mediated organ damage or symptoms suggestive of pre-eclampsia. In all other cases, the treatment threshold is ≥150/95 mmHg during pregnancy [16, 27]. Both American and European guidelines recommend methyldopa, nifedipine or labetalol when treating hypertension in pregnancy [15, 16, 27]. The scientific basis for treatment benefits of hypertension in pregnancy is, however, decidedly weaker than that in non-pregnant adults. Recently, a large, randomized, controlled trial which compared a blood pressure target of <140/90 mmHg to that of <160/105 mmHg in pregnant women with preexisting hypertension showed a lower risk of maternal, pre-eclampsia related outcomes for the intervention group (mean blood pressure 130/79 mmHg), compared to the control group (mean blood pressure 133/82 mmHg) [60]. Furthermore, there was no difference in fetal safety outcomes, which could serve as a compelling argument for more active antihypertensive therapy in pregnant women with preexisting hypertension. Whether the results from this study will impact future guideline-recommended treatment targets for pregnant women with preexisting hypertension remains to be seen.

## Differences in hypertension prevalence and blood pressure control

In population-based samples, hypertension is more common among men; the global, age-standardized prevalence of hypertension has been estimated at 32% in women and 34% in men [61]. Women also displayed higher rates of both hypertension treatment and hypertension control than men did in all regions of the world in these data, which did not include participants who were 80 years or older [61].

In samples from real-world, primary-care data, however, diagnosed hypertension was consistently more prevalent among women, with women constituting slightly more than half (52–58%) of patients in large, recent studies from industrialized countries [11, 62–67]. This discrepancy between population-based data and real-world data may be in part due to differences in health-care seeking behavior between men and women. Women are, overall, more likely to seek healthcare [68]. Increased exposure to healthcare, consequently, is likely to result in a higher likelihood of detecting and diagnosing hypertension. Indeed, previous work also shows that women more often receive a diagnosis of hypertension than men do in primary care [69]. The higher prevalence of hypertension among women in primary care may also be a consequence of the female population being slightly older than their male counterpart and hypertension becoming more prevalent with increasing age [70]. Real-world data, naturally, also include patients who are 80 years or older, among which hypertension is highly prevalent. For instance, data from the Region of Västra Götaland, Sweden, indicate that nearly half of the population have an established diagnosis of hypertension diagnosis at age 80 [11]. In contrast to population-based samples, controlled blood pressure is generally seen in higher frequency among men than in women in real-world data [65]. This may be a consequence of manifest cardiovascular disease being more prevalent among the male hypertensive population, with more ambitious antihypertensive treatment as a result. However, the same pattern was also evident in European survey data from patients with treated hypertension, but without manifest cardiovascular disease, who were managed in primary care. In that study 50.5 % of women and 56.6% of men attained a controlled blood pressure of <140/90 mmHg [71].

### Results on hypertension prevalence and blood pressure control from QregPV

**Fig. 1:**
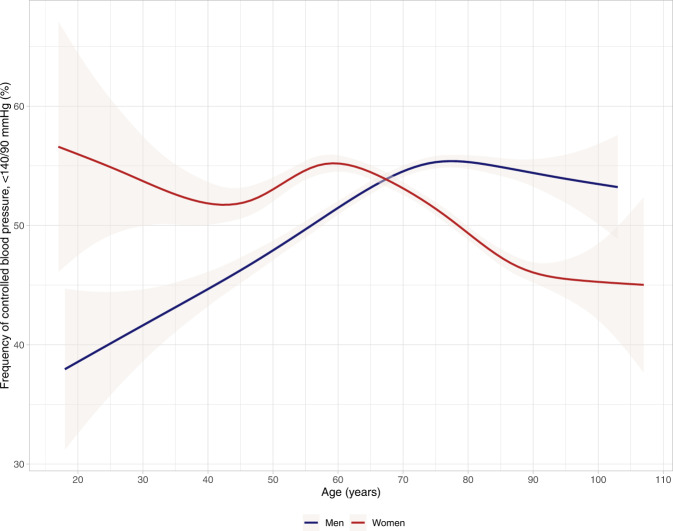
Frequency of controlled blood pressure, <140/90, among men and women, by age. The shaded, gray areas represent the 95% confidence interval.

**Fig. 2:**
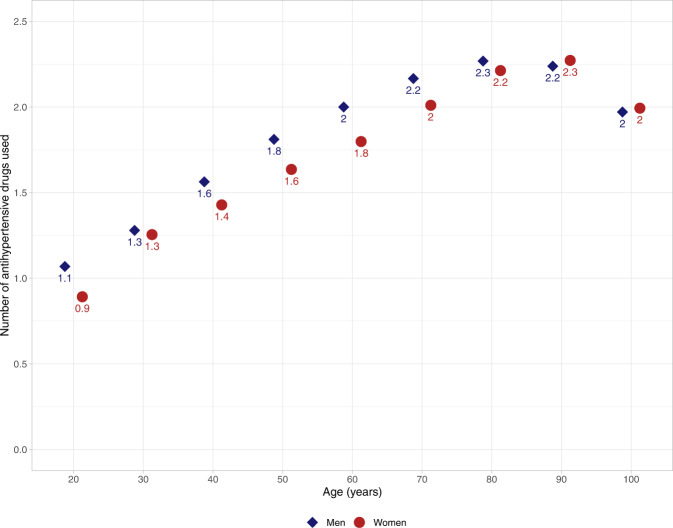
Sex differences in number of antihypertensive drugs used. Mean number of antihypertensive drugs used by men and women, by age.

For this article, we gathered original data from QregPV, a quality register for primary care. The study population comprised all 229 864 living patients with diagnosed hypertension in primary care in the Swedish region of Västra Götaland (population 1.7 million) in 2017. Hypertension was more prevalent among women, who constituted 51.4% of the study population. They were also slightly older than their male counterparts, 71.1 vs 68.4 years. See Table 2. We found that 51.7% of women and 53.3% of men attained a blood pressure <140/90 mmHg. On closer scrutiny of blood pressure control by age in QregPV, however, it is apparent that women attain higher rates of controlled blood pressure than men do until their sixties, whereafter the situation is reversed and men exhibit better blood pressure control, see Fig. 1. Women aged 80 years or older comprised 27.3% of all women with hypertension in QregPV, whereas the respective figure for men was 18.3%. Older women thus comprise a sizeable portion of patients with hypertension in general and of women with hypertension in particular. It follows from the blood pressure control patterns described above, that any analysis which does not include the entire age spectrum of patients with hypertension is bound to overestimate blood pressure control in women and underestimate it in men. This highlights a key difference between real-world data and population-based study data, where the latter are less likely to include the oldest patients with hypertension, who more often are women. This blood pressure control pattern, decreasing in women and increasing in men as they age, might be expected to be ensued by an increase in use of antihypertensive drugs in women and a corresponding decrease in men. This is not the case in our data, however, which show lower or similar numbers of used antihypertensive drugs for women, compared to men, throughout the entire age spectrum. See Fig. 2. Worse blood pressure control in older women, compared to men, is thus not accompanied by higher use of antihypertensive drugs. Whether this is a result of differences between men and women in comorbidities, predisposition for adverse drug effects or therapeutic inertia is debatable. In multivariable regression analysis adjusted for blood pressure, age and comorbidities, female sex was significantly associated with a lower use of antihypertensive drugs (*p* < 0.001) in our data. Comorbidities alone, thus, do not seem to explain the difference in numbers of antihypertensive drugs used.

**Table 2:** Characteristics and drug treatment of the study population, 229 864 women and men with hypertension in QregPV.

	Women	Men
Number (%)	118,252 (51.4)	111,612 (48.6)
Age (years)	71.4	68.6
IHD	13%	21%
Diabetes	21%	30%
SBP (mmHg)	137.0	136.1
LDL-C (mmol/L)	3.2	2.9
Smokers	13%	12%
Antihypertensive drugs per person (*n*)	2.0	2.1
ACEi	28%	37%
Alpha blocker	0.8%	1.8%
ARB	39%	39%
Beta blocker^a^	44%	43%
CCB	39%	43%
Labetalol	0.2%	0%
Loop diuretics	15%	13%
MRA	4.4%	4.5%
Thiazide	30%	28%
Other antihypertensives^b^	0.6%	0.5%
Statin	38%	50%

*IHD* ischemic heart disease, *SBP* systolic blood pressure, *LDL-C* low density lipoprotein cholesterol, *ACEi* angiotensin-converting enzyme inhibitor, *ARB* angiotensin-receptor blocker, *CCB* calcium-channel blocker, *MRA* mineral-receptor antagonist, *QregPV* quality register for primary care (“primärvård” in Swedish) in the Region of Västra Götaland, Sweden.

^a^Labetalol not included.

^b^Comprises centrally acting adrenergic blockers (methyldopa, clonidine, moxonidine), hydralazine, amiloride and renin inhibitors.

## Differences in drug class use

Despite the lack of a difference in effect on blood pressure and salient cardiovascular endpoints, antihypertensive drug class use differs between men and women in real-world data [2]. Thiazide use has been decidedly more frequent in women whereas ACEi and CCB have been used more in men [2, 62, 63, 65, 72, 73]. Previous work has shown that the differences in drug class use remains after adjusting for age and comorbidities [65].

### Results on drug class use from QregPV

In our data from 229,864 patients with diagnosed hypertension from 2017, the above-mentioned patterns of drug-class use are still evident. However, the thiazide discrepancy was only 2% and smaller than that for CCB (4%) and ACEi (9%), see Table 2. Alpha-blocker use was low overall, but roughly twice as prevalent in men (1.8%) compared to women (0.8%). ARB use was the same in both men and women (39%). Although women were older and had higher levels of systolic blood pressure and LDL cholesterol, they used slightly fewer antihypertensive drugs (2.0 vs 2.1) and had a lower use of statins. Men, on the other hand, displayed distinctly higher prevalence rates of ischemic heart disease and diabetes, conditions which incite treatment decisions in primary care.

**Fig. 3:**
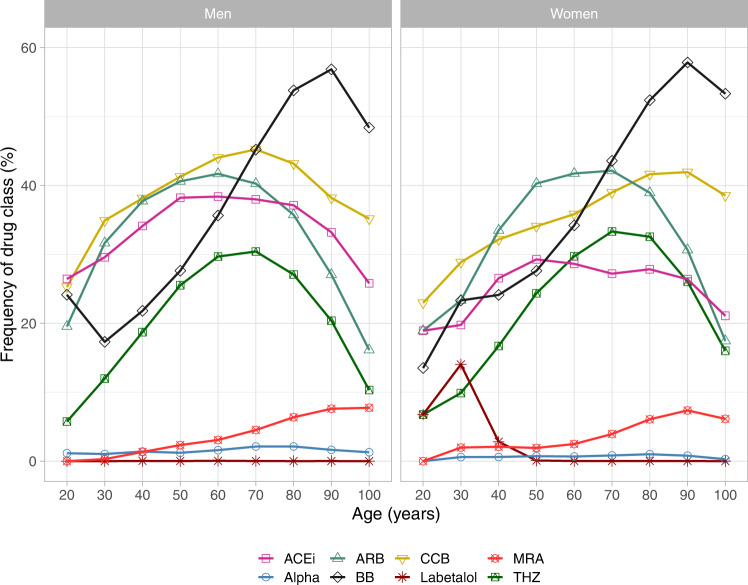
Age-specific use of antihypertensive drug classes in men and women. ACEi angiotensin-converting enzyme inhibitor, Alpha alpha blocker, ARB angiotensin-receptor blocker, BB beta blocker, CCB calcium-channel blocker, MRA mineral-receptor antagonist, THZ thiazide diuretic.

All drug classes used in hypertension fell off steeply in use after age 80, except for beta blockers and MRA, in both men and women, see Fig. 3 (interactive version available as a supplementary figure online). There was a distinct spike in labetalol use in women in ages 25–35 which is missing in men, and which can be attributed to its role in treating preexisting hypertension in women who are pregnant or pursuing pregnancy. In contrast, most women who develop *de novo*, gestational hypertension during pregnancy are managed in specialized, maternal care and are thus not encompassed in these data. Unfortunately, we lack specific data on the use of methyldopa, but it is our experience that it is an exceedingly rare treatment choice outside specialized, maternal care.

## Closing remarks

The last part of this review has highlighted how estimates of hypertension prevalence, control and drug treatment in men and women differ between large population-based studies and real-world data from routine clinical practice. In population-based studies, men have higher blood pressure, less treatment and worse blood pressure control; in real-world data the situation is reversed and women have higher blood pressure, less treatment and worse blood pressure control. As mentioned, this can be largely explained by the omission of the oldest patients, those 80 years of age or older, in the population-based studies. These older individuals comprised more than 20% of all patients with hypertension in our data from routine primary care, where patients 80 years or older are not omitted. The majority of these older patients are women and, as shown in Fig. 1, they have worse blood pressure control than their male counterparts. Hypertension treatment in patients aged 80 or older can be challenging, but both randomized controlled trial and meta-analysis data support that blood pressure control is beneficial even in this age stratum [74, 75]. In original data in this article, we also demonstrate that female sex is a significant predictor of less intensive antihypertensive treatment, a finding that warrants further exploration in future research.

As clinicians, we are armed with a plethora of proven drugs to combat hypertension and its detrimental effects on the cardiovascular system. The differing properties of these drugs can be harnessed to synergistically treat comorbidities both within and outside of the cardiovascular realm. Examples of the former, such as beta blocker treatment in heart failure and ischemic heart disease, are well known. Extracardiac treatment synergies are, perhaps, less obvious. For example, patients with hypertension and a history of episodic migraine and osteoporotic fractures might benefit doubly from treatment with angiotensin-receptor blockers, which decrease migraine episode recurrence, and thiazides, which may decrease fracture risk. Another example of treatment synergy from Table 1 would be using MRAs in patients with resistant hypertension and liver cirrhosis. When we are equipped with knowledge and understanding of the diverse properties and applications of our drugs, we can tailor the antihypertensive therapy of our patients to better fit their needs and preferences.

The higher use of antihypertensive drugs in men found in our primary-care data could be a result of their higher cardiometabolic comorbidity and consequent cardiovascular risk, but it is unlikely to be the sole explanation for the prescription discrepancy. For example, it is noteworthy that ARB, a drug class with an adverse event profile comparable to placebo, was used by 39% of both men and women, which makes it the most gender-equal drug in this analysis. Consequently, it is conceivable that part of the difference between men and women in both specific drug use and in overall number of antihypertensive drugs used per person is attributable to perceived adverse effects. It stands to reason that women are less inclined to tolerate ACEi when they are more likely to develop cough from the treatment than men are [6]. In the same vein, thiazide-related erectile dysfunction is likely to yield a swift drug discontinuation in many men [37–39]. Sex-related predisposition to adverse effects from antihypertensive drugs may thus affect prescription patterns. Conversely, thiazides may be a preferrable treatment for patients with osteoporosis, which predominantly are women, because of their suggested fracture-reducing effect [28, 29]. This, too, can contribute to sex-related prescription differences.

In addition to women being more susceptible to adverse effects from drug treatment, data from the AusHEART study also showed that their cardiovascular risk is underestimated [76]. Taken together, these factors may contribute to undertreatment of cardiovascular risk factors in women. A systematic, individualized risk assessment in all patients with hypertension – regardless of sex and presence of manifest cardiovascular disease – is thus warranted to avoid biased risk assessment and underestimation of cardiovascular risk and to guide treatment to prevent unnecessary cardiovascular disease. The recently released 2021 ESC Guidelines on cardiovascular disease prevention provide an excellent aid in such systematic risk assessment [77]. Men and women face different health challenges throughout their lives, which may necessitate different utilization of pharmacologic therapy. It is therefore both expected and reasonable for the treatment of hypertensive women to be different to that of hypertensive men. It should be noted, however, that whereas different treatment is acceptable, insufficient treatment is not.

## Supplementary information

Supplementary figure

Supplemental figure legend
