# Supplementary material for: Hypertension: sex-related differences in drug treatment, prevalence and blood pressure control in primary care
Source: J Hum Hypertens. 2023 Jan 19;37(8):662–70. doi: 10.1038/s41371-023-00801-5 (PMC10403353; doi:10.1038/s41371-023-00801-5)
Supplement: Supplementary file 2 — Supplemental figure legend [file 41371_2023_801_MOESM2_ESM.docx]

### Supplemental figure legend

**Interactive online version of figure 3. Age-specific use of antihypertensive drug classes in men and women.** When placing the cursor on a graph, the age-span and percentage of patients in that age-span who use the hypertensive drugs are shown. ACEi denotes angiotensin-converting enzyme inhibitor; Alpha, alpha blocker; ARB, angiotensin-receptor blocker; BB, beta blocker; CCB, calcium-channel blocker; MRA, mineral-receptor antagonist; THZ, thiazide diuretic
